# Supplementary material for: 16S rRNA gene sequencing reveals effects of photoperiod on cecal microbiota of broiler roosters
Source: PeerJ. 2018 Feb 22;6:e4390. doi: 10.7717/peerj.4390 (PMC5825889; doi:10.7717/peerj.4390)
Supplement: Supplemental Information 2 [file peerj-06-4390-s002.docx]

| Item | Value |
| --- | --- |
| Ingredient, g/kg |  |
| Corn | 672.65 |
| Soybean meal（43%） | 127 |
| wheat bran | 150 |
| Soybean oil | 10 |
| Alimet | 1.35 |
| Powder | 13 |
| CaHPO_4_ | 16 |
| Premixture**^a^** | 10 |
| Calculated nutrient composition (fresh basis) |  |
| Crude protein, g/kg | 13.51 |
| Ether extract, g/kg | 3.89 |
| Crude fiber, g/kg | 3.09 |
| Methionine + cysteine, g/kg | 0.59 |
| Calcium, g/kg | 0.91 |
| Available phosphorus | 0.45 |
| ASH,g/kg | 5.24 |
| AME, MJ/kg | 2793.39 |
